# Review: can diet influence the selective advantage of mitochondrial DNA haplotypes?

**DOI:** 10.1042/BSR20150232

**Published:** 2015-12-22

**Authors:** J. William O. Ballard, Neil A. Youngson

**Affiliations:** * School of Biotechnology and Biomolecular Sciences, The University of New South Wales, Sydney, NSW 2052, Australia; † Department of Pharmacology, School of Medical Sciences, UNSW Australia, Sydney, NSW 2052, Australia

**Keywords:** diet, epigenetics, flavin-adenine dinucleotide (FAD), mitochondria, retrograde response, selection

## Abstract

This review explores the potential for changes in dietary macronutrients to differentially influence mitochondrial bioenergetics and thereby the frequency of mtDNA haplotypes in natural populations. Such dietary modification may be seasonal or result from biogeographic or demographic shifts. Mechanistically, mtDNA haplotypes may influence the activity of the electron transport system (ETS), retrograde signalling to the nuclear genome and affect epigenetic modifications. Thus, differential provisioning by macronutrients may lead to selection through changes in the levels of ATP production, modulation of metabolites (including AMP, reactive oxygen species (ROS) and the NAD^+^/NADH ratio) and potentially complex epigenetic effects. The exquisite complexity of dietary influence on haplotype frequency is further illustrated by the fact that macronutrients may differentially influence the selective advantage of specific mutations in different life-history stages. In *Drosophila*, complex I mutations may affect larval growth because dietary nutrients are fed through this complex in immaturity. In contrast, the majority of electrons are provided to complex III in adult flies. We conclude the review with a case study that considers specific interactions between diet and complex I of the ETS. Complex I is the first enzyme of the mitochondrial ETS and co-ordinates in the oxidation of NADH and transfer of electrons to ubiquinone. Although the supposition that mtDNA variants may be selected upon by dietary macronutrients could be intuitively consistent to some and counter intuitive to others, it must face a multitude of scientific hurdles before it can be recognized.

## INTRODUCTION

The factors that maintain genetic and phenotypic variation within natural populations have long interested evolutionary biologists. Is this variation neutral and governed by random factors? Is it transient because of selection for or against particular alleles? Or, alternatively, does selection maintain variation? Biologists refer to this last case as balancing selection [1–3]. Balancing selection models grew out of ideas meant to account for the high, and sometimes extreme, levels of polymorphism in many species [4]. The mechanisms include spatial or temporal habitat heterogeneity, heterozygote advantage and negative frequency-dependent selection among others. Although differing in details, these mechanisms share the feature that whether an allele is beneficial or detrimental is conditional in some way. An allele cannot be described as advantageous or deleterious, except in a particular context. The goal of the present review is to explore the potential for mitochondrial functions to influence the selective advantage of specific mtDNA haplotypes through dietary change. This dietary change may be seasonal, result from population expansion or longer-term changes in climate for example.

For many years, mitochondria were viewed as a semi-autonomous organelle and required only for cellular energetics. This view has been largely supplanted by the concept that mitochondria are more than an evolutionary hitchhiker but rather are fully integrated into the cell and essential for normal cellular functioning [5,6]. Mild mitochondrial stress that can be triggered by any of a variety of insults results in a broad and diverse cytosolic and nuclear response has been termed mitohormesis [7]. Although varied, this response appears to induce a wide-ranging cytoprotective state resulting in long lasting metabolic and biochemical changes. Remarkably, rather than being harmful, these changes may increase evolutionary potential and decrease susceptibility for disease. Although the exact response depends, in part, on the specific mitochondrial perturbation, in general, the transcriptional response results in a reconfiguration of metabolism, allowing for the production of essential intermediates such as glutamate and increasing glycolytic production of ATP [8].

mtDNA is located in the mitochondria and is packaged into DNA–protein assemblies that facilitate its involvement in cellular metabolism [9]. In most multicellular organisms, mtDNA is circular, covalently closed and double stranded [but see 10,11]. Importantly, proteins derived from mtDNA reside in a figurative sea of proteins encoded by the nuclear DNA (nDNA) and imported into the mitochondrion, which has given us insight into the evolutionary history of the organelle.

In the present review, we explore whether diet has the potential to differentially influence mitochondrial bioenergetics such that the frequency of mtDNA haplotypes changes due to positive selection. The macronutrient balance of metazoans is frequently seasonal and may include periods of prolonged starvation. Diet and the relative proportions of macronutrients may also change when species colonize new habitats or be altered with longer term biogeographic changes. Simpson and Raubenheimer [12] predicted that animals are under strong selection for the ability to regulate the ratios of macronutrients eaten, through choosing foods that are balanced with respect to requirements or eating appropriate proportions of nutritionally complementary foods when consuming a mixed diet. However, optimal macronutrient intake is often not possible in circumstances when vagility is low or resources are limiting. Naturally occurring intra-specific differences in bioenergetics and ATP production have been documented in a wide range of organisms including snakes [13], frogs [14], birds [15], flies [16], mice [17] and humans [18]. This variation can have important evolutionary consequences. Salin et al. [19] suggest that the mitochondrial oxidative phosphorylation (OXPHOS) efficiency is an important proximate factor for constraining life history trajectories whereas Hill [20] proposes that efficiency of cellular respiration, as a product of mitochondrial function, underlies the associations between ornamentation and performance for a broad range of traits. Reducing the rates of oxygen consumption and ATP production may also induce a decrease in growth rate and body size [21], which is frequently being positively correlated with fecundity [22].

In the present study, we aim to integrate information from a range of studies considering organisms in the wild, the farmhouse and the laboratory and glean evidence from a range of systems including mammals and fish. *Drosophila* is one focus because they have a natural history and are an established laboratory model. We also consider the role that epigenetics could have in this process. The importance of epigenetics in the regulation of development is well-established, but it is now becoming apparent that it is also a prominent player in the mediation of gene-environment interactions. To clarify the cellular and biochemical aspects of mitochondrial bioenergetics, we also touch on aspects of cancer biology and human disease.

In the article, we briefly review mitochondria and the mitochondrial interactome. In the present study, we define the mitochondrial interactome as the whole set of molecular connections that occur within mitochondria. We then consider how diet can influence the ratio of macronutrients entering the electron transport system (ETS). Next we investigate the potential for specific mtDNA mutations to influence the dynamics of this system. Finally, we conduct a case study of ETS complex I where we integrate mtDNA and dietary data. Evidence from these studies suggests that diet has the potential to influence the bioenergetics of specific mtDNA haplotypes and this may in turn shape the frequency of these haplotypes in nature. We fully acknowledge our understanding of the mechanistic links between mitochondrial metabolism, diet, health and fecundity are far from complete. One difficulty in making these connections is that the mitochondria must function in a wide range of cellular environments. Further, distinct selective forces may be operating in specific tissues, cells and life history stages [23]. Still, extension of highly controlled studies and logistic equations [24] to the natural environment would seem to be within sight.

## MITOCHONDRIA AND THE MITOCHONDRIAL INTERACTOME

Over the past decade, biochemical, cellular and physiological studies have revolutionized the way we think about mitochondria. In large part, this has come from studies of human diseases as well as the decline in mitochondrial function with advancing age. We now know that mitochondria are dynamic organelles capable of changing size and connectivity, they house the cellular apoptotic machinery to control cell survival, they elicit robust signalling responses that enable the cell to respond to energetic demands and they can influence rates of development and reproduction [25]. There is also strong evidence of direct mitochondria–endoplasmic reticulum communication facilitated by the physical interaction of their membranes, which enables calcium and lipid transfer between organelles and can also act as platforms for signalling [26]. Alas, it also seems true that advances in our understanding of the cell biology and biochemistry of the organelle continue to remain unincorporated into contemporaneous thinking of the bioenergetics of mitochondrial metabolism [25,27] and the evolutionary dynamics of mtDNA [28]. In this section we review the basic functions of mitochondria. We then consider the relationship between the mtDNA and the mitochondrial interactome to co-ordinate the proper functioning of mitochondria.

### Mitochondria

**Figure 1:**
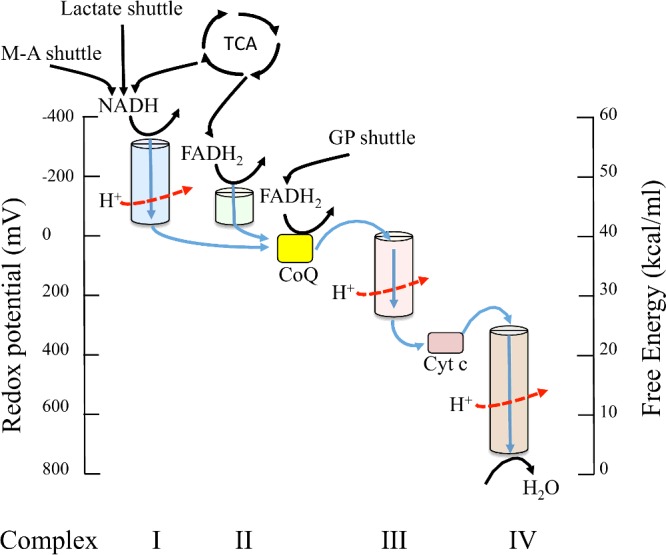
Redox potentials, and the corresponding free energy levels, of electron carriers in the respiratory chain (complex I–IV) Red arrows show proton pumping. The lowest potential is found with complex I in keeping with its position at the start of the transport chain. The malate–aspartate (M-A) and lactate shuttles in addition to the TCA cycle provide NADH and this is reduced to NAD + H^+^. With complex II, the potentials of both entry and exit points must fall into the narrow interval between FADH_2_ and coenzyme Q, which means that very little energy is released as electrons traverse this complex. The TCA cycle provides FADH_2_ and this is reduced to NAD + H^+^. The glycerol 3-phosphate shuttle (GP) shuttle provides FADH_2_ to coenzyme Q (CoQ) between complexes III and IV via cytochrome *c* (cyt c). Such minor steps in redox potential suffice to ‘jog’ the electrons along, but they are too small to contribute to proton pumping. The redox potential increases continuously along the respiratory chain to reach its highest value at oxygen, which therefore has the highest affinity for the electrons and gets to keep them. Reduced oxygen, which recombines with protons to yield water, is the end product of respiration.

Mitochondria perform multiple metabolic functions, most notably the generation of energy from proteins, carbohydrates and fatty acids [29,30]. Briefly, mitochondria use electrons harvested from oxidizable substrates and O_2_ as a final electron acceptor to build up a proton-motive force by pumping protons from the mitochondrial matrix into the intermembrane space (Figure 1). The subsequent backflow of protons to the matrix across complex V (ATP synthase) of the inner membrane drives the synthesis of ATP. This whole process is referred to as OXPHOS. Hence, one main function of mitochondria is to couple respiration (i.e. oxygen consumption) and substrate oxidation to ATP synthesis [31,32]. Indeed, approximately 90% of oxygen consumption in the basal state is mitochondrial and 80% of this is coupled to ATP production. Importantly, in the context of the present review, mitochondria also provide signalling molecules as well as metabolites for anabolic processes such as *de novo* synthesis of fatty acids and gluconeogenesis.

The efficiency of ATP production by mitochondria in a specific environment is likely to be one parameter that influences the success of an organism harbouring a specific mtDNA haplotype. Studies in endotherms that focused on feed efficiency in poultry and livestock species have suggested that the efficiency of mitochondrial metabolism may affect the capacity of an animal to efficiently convert energy contained in food into body mass and the rate of growth [15]. Traits associated with metabolism have also been shown to be highly differentiated between closely related species and perhaps even intimately involved with speciation [33]. For example, lake whitefish have independently evolved ‘dwarf’ and ‘normal’ sympatric species pairs that exhibit pronounced phenotypic and genetic divergence across multiple lakes in North America. Dwarfs have evolved from the ancestral benthic form and allocate a greater proportion of their energy budget to metabolism, exhibiting a lower bioenergetic conversion efficiency compared with normal whitefish. Evans and Bernatchez [34] examine the transcription of mitochondrial genes in whitefish species pairs from different lakes and observed OXPHOS gene up-regulation in dwarf whitefish. Based on these data, the author's posit that OXPHOS up-regulation may be involved in meeting the enhanced energetic demands of dwarf whitefish.

One metric of mitochondrial function is the ADP/O ratio. It is calculated as the moles ADP phosphorylated per mole oxygen atom consumed. Differences in ATP production may affect development times and rates of reproduction [25], which may in turn influence the frequency of mitochondrial haplotypes in nature. Methods for measuring the ADP/O ratio in isolated mitochondria were developed more than 50 years ago by Chance and Williams [35]. Subsequently, protocols have been developed that allow the use of permeabilized cells or tissues as well as homogenized tissue to analyse mitochondrial functions [36].

Several parameters influence the ADP/O ratio. These include the diet of the organism and the substrate used in biochemical studies [25,32]. Diet composition can also have an indirect effect on the ADP/O ratio, since it can affect the phospholipid properties of the inner mitochondrial membrane and potentially contribute to proton leak [37,38]. Reductions in the ADP/O ratio may result from fewer protons pumped by the electron-transport-chain complexes for each electron pair transferred, less ATP made by the ATP synthase for each proton driven through it (collectively called slip reactions), uncoupling proteins whose function is to catalyse a regulated inducible proton conductance and leak of protons across the membranes [32]. In the present review, we do not consider indirect effects though there has been extended debate on its importance particularly in humans [39–43]. In the following section, we review the basic structure of mtDNA because it can influence mitochondrial bioenergetics.

### Mitochondrial interactome

The two strands of mtDNA are differentiated by their nt content, with a guanine-rich strand referred to as the heavy strand and a cytosine-rich strand referred to as the light strand. The heavy strand encodes 28 genes and the light strand encodes nine genes for a total of 37 genes. Of the 37 genes, 13 are for proteins (polypeptides), 22 are for tRNA and two are for the small and large subunits of rRNA. There is also a region around the origin of replication that is called the D-loop region in mammals and the A + T-rich region in many invertebrates.

The 13 mitochondrial protein coding genes in metazoans encode subunits for four of the five complexes. Mammalian complex I, has seven mtDNA-encoded subunits (ND1, ND2, ND3, ND4, ND4L, ND5, ND6) and 37 nuclear-encoded subunits [44]. Complex II is composed of four nuclear-encoded protein subunits. Complex III has the single cytochrome *b* mitochondrial subunit and 10 nuclear-encoded subunits. Complex IV has three mtDNA (COI, COII, COIII) and typically 10 nuclear-encoded subunits [but see 45]. A unique feature of complex IV is the presence of subunits with tissue-specific isoforms [46]. Complexes I, III and IV form higher order structures called ‘supercomplexes. It is hypothesized that these supercomplexes enhance respiration, possibly by decreasing the distances among active sites or improving the stability of complexes [47]. Mutations in any complex have the potential to influence dynamics of the ETS but codon usage patterns seem to be determined principally by complex context-dependent mutational effects [48].

Complex V is the final subunit of the ETS. It has two mitochondrial- (ATP6, ATP8) and nine nuclear-encoded subunits. Complex V uses the concentration difference of protons between both sides of the inner membrane of the mitochondria to drive the force behind ATP synthesis (Figure 1). It consists of two regions: the F_0_ region within the membrane and the F_1_ portion inside the matrix of the mitochondria. If the F_0_ proton-translocating domain of ATP synthase is not properly connected to the F_1_ sector of this enzyme, protons will leak through the inner membrane [49].

Genes from both genomes encode the components of the mitochondrion's ribosome. Ribosomal proteins are encoded in the nuclear genome where they are synthesized on cytoplasmic ribosomes and then imported into the mitochondria. There, they are assembled with the two mtDNA-encoded rRNA's in the mitochondrion and are responsible for translating the 13 mitochondrial mRNAs. Mutations in rRNA's are known to decrease mitochondrial protein synthesis affecting all proteins similarly [50].

Mitochondrial tRNA's play a critical role in the translation of mtDNA-encoded proteins in the mitochondrial matrix, where they interact with a nuclear-encoded aminoacyl-tRNA synthetase. The aminoacyl-tRNA synthetase is responsible for ‘charging’ the appropriate tRNA with the correct amino acid [51]. Once charged, the tRNA brings the amino acid to the ribosomal complex for integration into a growing polypeptide chain based on complementary anti-codon base pairing. Importantly, tRNA mutations are the most common examples of human mitochondrial disease. These genes compose less than 10% of the mitochondrial genome but are responsible for more than half of the identified human disease mutations (http://mitomap.org/MITOMAP).

The D loop in mammals, or the A + T rich region in many invertebrates, has often been considered to be without biological function. However, mounting evidence suggests this is not the case as it is the region where mitochondrial replication and transcription initiate and it is the major site of transcriptional regulation [52,53]. Mercer et al. [54] conducted an in-depth study of the mitochondrial transcriptome and observed complex DNaseI footprint profiles within this region, including those associated with TFAM (transcription factor A, mitochondrial)-binding sites, light strand transcription initiation, transcription termination and sites of RNA-to-DNA transitions.

Due to the dual genetic origin of the ETS, it is critical to co-ordinate the expression of its mtDNA- and nDNA-encoded components. Mitochondrial-nuclear genetic interactions play important roles in modulating development and reproduction and these epistatic interactions are further modified by diet. More generally, these findings illustrate that gene-by-gene and gene-by-environment interactions are not simply modifiers of key factors affecting mitochondrial bioenergetics, but these interactions themselves are the very factors that underlie important variation in this trait [55,56]. Although the focus of the present review is to consider the potential for specific mtDNA types to be selected, it is clear that the nuclear background will influence the selective advantage of any specific haplotype both through mitonuclear interactions [57,58] and through epigenetic effects [59,60]. The nuclear genome can also be epigenetically modified by diet [61] and thereby facilitate or impede the selective advantage of a particular mtDNA haplotype. With these caveat's having been issued, it still remains possible that mtDNA differences can influence organismal fitness in a broad range of nuclear genetic backgrounds. Christie et al. [62] examined the fitness of two common and eight rare mtDNA haplotypes in *Drosophila subobscura* and found that haplotype VIII likely has a positive effect upon the fitness of its hosts independent of the nDNA background. Ballard and James [63] employed population cage studies to show that the three *Drosophila simulans* mtDNA haplotypes have unequal fitness in the laboratory and the frequency of the haplotypes correlated with their known worldwide distribution. James and Ballard [64] controlled the nuclear genome of *D. simulans* by backcrossing and found repeatable differences in larval and adult life-history traits that were caused by mtDNA.

In this section, we reviewed the basic functions of mitochondria and considered how mtDNA and nDNA combine together to ensure how the mitochondria function. We have not, however, considered how the genomes co-ordinate their expression patterns to ensure optimal mitochondrial functioning. This is achieved via the co-ordination of mitochondria-to-nucleus signalling pathways, known as the retrograde response or the retrograde pathway in yeast (RTG) [65]. The following section considers the retrograde response.

## RETROGRADE RESPONSE

**Figure 2:**
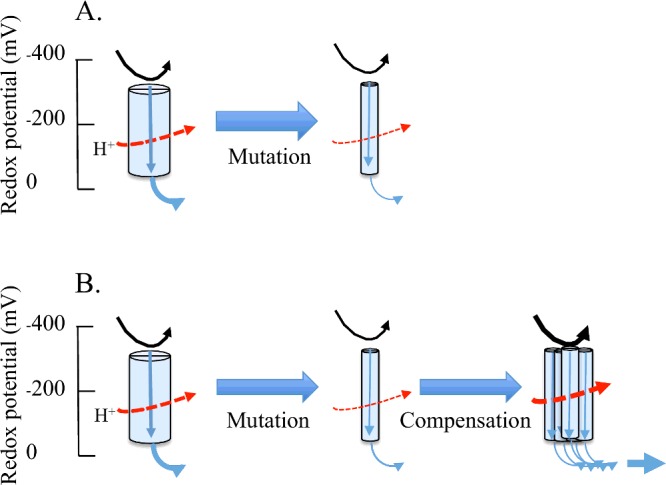
Cartoon depicting the potential influence of deleterious and slightly deleterious mutations in complex I (**A**) A deleterious mutation may be expected to restrict the flow of electrons and reduce the potential for proton pumping. In this case, it is diagrammatically represented by the reduction in circumference of the complex. This is expected to cause a reduction in fitness and possibly disease. (**B**) In this case, the mutation causes a reduced flow of electrons but compensation/ overcompensation enables electron flow to be restored. This compensation may occur through a retrograde response to the nuclear genome which effectively increases the number of each inefficient complex. This may result in a short term increase in fitness by increasing ATP production.

Subject to resource availability in a healthy young organism, the deleterious consequences of some mtDNA mutations may be overcome by cellular and biochemical retrograde responses. Biochemically, this occurs through the metabolic manipulation of components of the ETS or by employing alternative biochemical pathways (Figure 2). Such retrograde responses can result in an adjustment of ATP and/or reactive oxygen species (ROS) production and lead to an increase in early fecundity [66]. The trade-off for these short compensatory responses may be a shortened lifespan [66]. In such cases, standardizing ATP and ROS levels by mtDNA copy number may be a useful strategy to determine the efficiency of a particular mtDNA haplotype.

The retrograde response adapts cells to changes in the functional state of mitochondria, such as respiratory defects, by mediating an assortment of cellular processes that include metabolic reconfiguration, nutrient sensing, aging and stress response pathways. In yeast, the RTG is regulated positively by Rtg1, Rtg2, Rtg3 and Grr1 and negatively by Mks1, Lst8 and two 14-3-3 proteins, Bmh1/2 [67–69]. In mammals and *Drosophila*, the underlying pathways have not been clearly established but the retrograde response has been observed and it would seem likely that signalling molecules are involved [70]. In a wide spectrum of metazoans, specific signalling pathways are modulated in part by metabolites controlled by the mitochondrion, including ATP, ROS and the NAD^+^/NADH ratio. In this section, we consider signalling molecules that may be involved in the retrograde response that communicates the biochemical and bioenergetic status of the organelle to the nucleus. In the present review, we do not consider mitochondrial membrane potential or the unfolded protein response as these have been elegantly covered elsewhere [7].

### ATP/AMPK signalling

The interaction between specific mtDNA mutations and substrates influences mitochondrial functions and this can alter processes that require ATP as well as ATP homoeostasis [71]. The AMP-activated protein kinase (AMPK) is a sensor of cellular energy status, expressed in essentially all eukaryotic cells as heterotrimeric complexes containing catalytic α-subunits and regulatory β- and γ-subunits. In mammals, AMPK is activated by increases in AMP/ATP or ADP/ATP ratios, which occur when cellular energy status has been compromised. AMP is well suited to be a primary signal to which the system responds. AMP levels are 100-fold lower than ATP in unstressed cells and an increase in ATP hydrolysis in stressed cells can result in a large fold increase in AMP.

ATP levels are well defended in cells. Zhang et al. [69] propose that the retrograde response mediates ATP homoeostasis by participating in a conserved negative feedback loop that responds to ATP levels to shut off ATP production when ATP is in excess. In yeast, the RTG pathway regulates the expression of genes encoding the first three Krebs cycle enzymes and activation of this pathway is expected to increase the metabolic flux into the Krebs cycle and ATP synthesis in mitochondria. When the level of cellular ATP reaches a certain threshold (3–4.5 mM), ATP releases Mks1 from Rtg2 to turn off the RTG. Together, these two processes help achieve cellular ATP homoeostasis.

In flies, a reduction in ATP production may lead to retrograde responses. A mutation in the complex IV gene cytochrome *c* oxidase Va has been reported to cause a 60% reduction in ATP production and this arrests the cell cycle in G_1_ through a pathway involving AMPK and p53 [72]. Ballard et al. [73] modelled a two amino acid deletion in the same complex and predicted it would lower cytochrome *c* oxidase activity. This prediction was observed, but unexpectedly, elevated levels of mRNA expression occurred for multiple genes encoding subunits of complexes I, III and IV suggesting a compensatory response to the deletion. This compensatory response caused young flies homozygous for the deletion to have a higher ADP/O ratio and increased fecundity but suffer from elevated levels of ROS and reduced survival [66]. We discuss the role of ROS as a signalling molecule in the next section.

### Balance of ROS

Payne and Chinnery [74] appraise the importance of ROS in organisms and come to a modern nuanced view that all ROS is not necessarily bad. Rather the key is probably the balance of ROS and scavenging in each subcellular location in which the molecules are acting, which determines the organelle and cellular health. Sublethal levels of ROS (<0.7 μM) can promote proliferation, genomic and epigenetic alterations, differentiation and survival in leukaemic cells; higher ROS levels (>1 μM) however can cause severe oxidative stress that leads to cell death [75]. Within mitochondria, most ROS is produced from complex I, complex III and glycerol 3-phosphate dehydrogenase [76]. However, when complex I and complex III are inhibited and succinate concentration is low, complex II in rat skeletal muscle mitochondria can generate superoxide or H_2_O_2_ at high rates [77]. Under very specific conditions *in vitro*, it has been estimated that between 1% and 2% of the total O_2_ consumed by mitochondria is converted into ROS by univalent reduction of oxygen to form superoxide [78]. However, previous estimates suggest that *in vivo* levels of ROS production may be at least an order of magnitude lower than earlier estimates [79,80]. Superoxide dismutase converts superoxide into hydrogen peroxide that is, in turn, reduced to water by glutathione peroxidase and/or catalase. Of these chemically reactive molecules, hydrogen peroxide is probably an important signalling molecule as it is lipid soluble and can freely cross membranes.

D'Autréaux and Toledano [81] argue that the chemical reactivity of ROS distinguishes it from other signalling molecules. They note low levels of hydrogen peroxide activate the p53 antioxidant response, whereas high levels trigger p53-dependent apoptosis through the induction of pro-oxidant activities. In mammals, the KEAP1–NRF2 complex constitutes the closest fit to a ROS receptor. The pathway plays a central role in hormesis by regulating the inducible expression of many cytoprotective genes in response to oxidative and electrophilic stresses [82].

Transcription factors of the class O forkhead box (FOXO) family are activated by hydrogen peroxide and induce either cell death or a quiescent cell state that is characterized by improved tolerance to oxidative stress [81]. FOXO activity is also regulated in response to hydrogen peroxide through acetylation by the cAMP responsive element-binding protein and deacetylation by the sirtuin SIRT1 [83]. Importantly, dFoxo only mediates the nutrient responsiveness for a subset of genes encoding mitochondrial proteins [84]. This implies that other transcription factors probably exist, but have not been described in *Drosophila*.

### NAD/NADH_2_ ratio

The NAD/NADH ratio plays a very important role in maintaining cellular redox homoeostasis. It could be considered as a cellular metabolic readout that allows for real-time monitoring of the metabolic state of a cell during pathophysiological changes [85]. Indeed, the functionality of mitochondrial metabolism is highly dependent on the maintenance of the organellar NAD pool and any mitochondrial processes depend on the universal coenzyme NAD or its phosphorylated counterpart NADP [86]. The redox reactions involve the reversible hydride transfer at the nicotinamide moiety of NAD(P), resulting in a switch between oxidized (NAD+, NADP+) and reduced (NADH, NADPH) forms of the nt. Critically, mitochondria usually contain a major portion of the cellular NAD content, with up to 70% of the cellular pool, depending on tissue and cell type [87]. In mammals, and probably other metazoans, NAD levels are influenced by intra-organellar biosynthesis of the molecule but may also be transported by shuttles [86]. We discuss these shuttles at greater length below.

NAD levels are modulated in response to nutritional and environmental stimuli including stress. For instance, it has been shown that calorie restriction enhances NAD levels in the liver, skeletal muscle, white adipose tissue and brain [88,89]. Fasting has also been shown to increase NAD levels to the same degree in the liver and skeletal muscle [88,90,91]. Some studies have even localized the increases in NAD levels generated by caloric restriction [88] and fasting [90] to the mitochondria. Mirroring the effect of low energy intake on NAD levels, high-fat diet feeding significantly reduces NAD levels in the liver and white adipose tissue [92]. These changes in NAD levels in response to energy intake appear to be caused by changes in nicotinamide phosphoribosyltransferase (NAMPT, the rate-limiting enzyme in the NAD biosynthetic pathway) expression. Fasting and caloric restriction induce NAMPT expression 2–3-fold in the liver and skeletal muscle [88,90,91].

In the next section, we consider epigenetic modulation of the mitochondrial and nuclear genomes by mitochondrial products. As it is central to cellular homoeostasis, any alteration of mitochondrial function will have downstream effects on countless processes, inevitably including changes to the epigenetic regulation of the nuclear genome. This can occur through a circuitous route: changes in mitochondrial metabolism are detected by cytoplasmic proteins that signal to transcription factors, which then recruit epigenetic modifiers and target them to specific regions of the genome. However, there are more direct routes whereby the actual products of the mitochondria are the molecules that will be incorporated into DNA or chromatin or are cofactors for the epigenetic modifier enzymes. These mechanisms highlight the finely-tuned and complex cross-talk between mitochondria and the nuclear genome.

## EPIGENETIC CROSS-TALK

Despite having been studied for over 40 years, the role and even the existence of epigenetic modifications on mtDNA remains poorly understood and controversial [93–95]. However, due to a renaissance of the field over the last 5 years, there is currently a strong case for the existence, if not yet the function of DNA methylation on mtDNA. The levels of mtDNA methylation have been shown to vary between different tissues and developmental stages, in disease-states, due to exposure to environmental pollutants and through modulation of the epigenetic machinery [94–100]. With regard to function, specific methylation changes have been found to correlate with differences in the mRNA levels of mtDNA-encoded transcripts but a direct, causative relationship has not been confirmed [94,97,98]. Over the next few years, we can expect a spotlight will focus on the potential for mtDNA methylation to contribute to various aspects of mitochondrial biology, including the effects of mtDNA sequence variation on organismal fitness.

In general, elevated levels of ATP and acetyl-CoA are converted into the epigenetic modification of genes through their use as donors of phosphate- and acetyl-groups for phosphorylation and acetylation of histones [101]. These epigenetic modifications serve to open chromatin and increase transcription of genes involved in metabolism and cell replication. Times of dietary stress lead to decreases in ATP and acetyl-CoA and the corresponding reductions in histone acetylation and phosphorylation, but will also increase the oxidation of NADH to NAD^+^. NAD^+^ is a coenzyme for the sirtuin class of histone deacetylases, which can reverse chromatin states from ‘open’ to ‘closed’ to repress genes.

Another important way in which mitochondrial output can directly influence epigenetics is through one-carbon metabolism. This process involves the intersection of the folate and methionine cycles. In the former cycle, folate is eventually converted into 5-methyltetrahydrofolate which then provides a methyl group to the latter cycle through the methylation of homocysteine, which is then converted via intermediates to *S*-adenosyl methionine (SAM) which is the main donor of methyl groups for histone and DNA methylation [102]. Mitochondrial metabolism contributes to the production of SAM as steps of the folate cycle can occur in the mitochondria and as SAM is composed of methionine and ATP.

**Figure 3:**
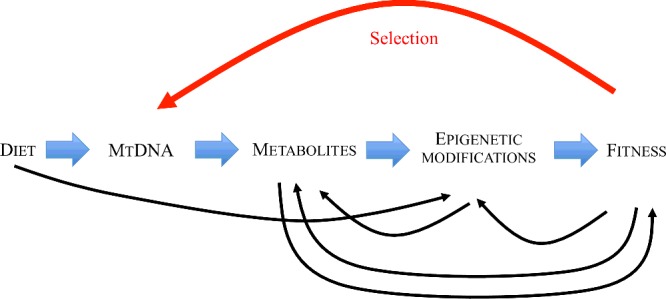
The potential role of epigenetics in the mediation of diet-induced shifts in the proportion of mtDNA haplotypes in a population Different dietary components, such as proteins, carbohydrates and fats, produce varying amounts of metabolites such as methyl-donors, ROS, NAD+, ATP, succinate and fumarate. mtDNA haplotypes influence the relative levels of these metabolites. Levels of these metabolites have been shown to alter epigenetic state directly through changes in gene expression or indirectly/independently of epigenetics with processes such as post-translational protein modifications in cell signalling (e.g. phosphorylation). Finally, ingestion of metabolites such as B vitamins can directly alter genomic epigenetic state without being processed by the mitochondria. The deeply inter-linked and complex nature of the components of the pathway are illustrated by the black arrows.

Epigenetics may also enable the persistence of (mtDNA-induced) gene expression changes. Exposure of humans, rodents and flies to starvation or high-energy (high fat, high sugar) diets, in early life or even in previous generations, can programme lifelong epigenetic changes that alter the expression of genes involved in processes that contribute to an organisms fitness [103–106]. In this way, epigenetics contrasts with the other molecular components that link mtDNA haplotype to fitness-altering gene expression changes, such as signalling proteins and transcription factors, which have been traditionally viewed more as responsive regulators of short-term changes in homoeostasis. A pathway displaying the potential role of epigenetics in mediating the shifts in the proportion of mtDNA variants in a population is presented in Figure 3.

In the next section, we consider how dietary macronutrients influence metabolism, ATP production and signalling by mitochondria. It is becoming increasingly clear that metabolism and cellular signalling are not just separate entities but rather are tightly linked. Biochemists have empirically determined mitochondrial ADP/O ratios from a wide range of taxa and found that these depend upon the nature of the substrates oxidized. Estimates, based on a combination of empirical data and theoretical considerations, reveal an ∼15% higher cellular ADP/O ratio when glucose is oxidized compared with fatty acid oxidation [32,107]. Translation of these cellular data to the whole organism is complex but has potential to give physiological and life history insight. For example, it was suggested that the requirement for less oxygen when oxidizing carbohydrate may be a possible mechanism facilitating hovering in humming birds at high altitude [108].

## NUTRIENTS CAN INFLUENCE METABOLISM AND SIGNALLING

**Figure 4:**
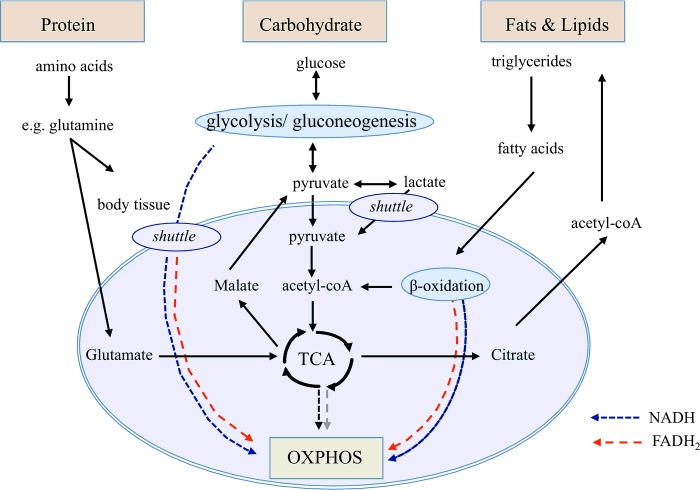
Nutrient metabolism Proteins: The first step in protein metabolism is to break it into its constituent amino acids. The second step is to break down amino acids into their constituent parts. One important amino acid is glutamine. Mitochondrial utilization of glutamine begins with a two-step conversion of glutamine to 2-oxogluterate, typically by glutaminase and glutamate dehydrogenase. 2-Oxogluterate can be either oxidized to succinate (standard TCA cycle) or reductively carboxylated (reverse TCA cycle) to isocitrate and then to citrate. Glutamine-derived malate can then be transported to the cytoplasm through the process of glutaminolysis to generate pyruvate and lactate. This reverse TCA cycle generates glutamine-derived citrate that can then be transported to the cytoplasm to generate acetyl CoA for anabolic processes such as fatty acid synthesis. Carbohydrates: Carbohydrates are made out of simple sugars such as glucose. The first step of carbohydrate metabolism is glycolysis. It is the metabolic process that converts glucose into two molecules of pyruvate. The second step of carbohydrate metabolism occurs in the mitochondria. Here pyruvate is oxidized to acetyl-CoA and CO_2_ by the pyruvate dehydrogenase complex in the mitochondria of eukaryotic cells and in the cytosol of prokaryotes. In a third step, when oxygen is present, the mitochondria will undergo aerobic respiration, which leads to the TCA cycle. Fat: Once the fatty acids have been transported to the mitochondrial matrix via the carnative pathway β-oxidation of fatty acyl-CoA occurs in a four step process. Assuming an even numbered carbon chain fully saturated fatty acid each cycle of β-oxidation produces one FADH_2_, one NADH and one acetyl-CoA (which produces one FADH_2_ and three NADH through the TCA cycle). This gives a total of two FADH_2_ and four NADHs inputs per cycle of β-oxidation or a FADH_2_/NADH ratio of approximately 0.5. But the very last pair of carbon atoms in a saturated fat already comprises acetate attached to CoA, so they can simply enter the TCA as acetyl-CoA (producing one FADH_2_ and three NADH).

In this section, we focus on the role of protein, carbohydrates and fats in mitochondrial functions and ponder how this might influence the frequency of mtDNA haplotypes (Figure 4). We also examine starvation as the ultimate dietary extreme. Here we take data from cellular studies and apply them to the whole organism. However, provisions need to be made in making such links. Specifically, we often do not know which process or processes in which tissue or tissues are rate limiting in terms of organismal development and reproduction. Certainly, we do not fully understand if cell-specific biases are being introduced. Disjunct remains between studies that occur at the cellular and organismal levels and studies in the laboratory and in nature. Unquestionably, studies diminishing the distance between the research domains are needed.

In principle, changing the relative ratios of dietary macronutrients will modify the point at which electrons enter the ETS and this has potential to influence mitochondrial metabolism. Consider, the redox cofactors NADH and FADH_2_. NADH and FADH_2_ enter the ETS at complex's I and II respectively (Figure 1). When complex I is the electron source, a total of 10 H^+^ are pumped to the intermembrane space (four from complex I, four from complex III, two from complex IV). When complex II is the electron source, there are no protons from complex I and there are a total of six H^+^. Since the production of ATP is not directly linked to each step in the transport of electrons, but rather by the gradient arising from pumping of protons during the electron transport, it is not necessary that an exact integral number of ATPs be produced per entry point. In fact, it appears that 2.5 molecules of ATP are produced for every oxygen atom reduced from complex I and 1.5 ATP from complex II. Levels of ROS output as a by-product of electron transport also differs between ETS substrates [76,109].

One approach that has been successfully employed to manipulate the ratio of macronutrients eaten by animals is called the geometric framework. The geometric framework was developed to explore how an animal solves the problem of balancing multiple and changing nutrient needs [12]. Lee et al. [110] employed the approach to determine how manipulating the protein/carbohydrate (P/C) ratio in the diet may influence female fecundity of *Drosophila*. The authors showed that a P/C ratio of 1:2 resulted in highest rates of early fecundity whereas a ratio of 1:4 resulted in greatest lifetime fecundity because flies lived longer on the latter diet. Solon-Biet et al. [111] used the same framework to investigate organismal and cellular functions in mice. Crucially, they observe the ratio of macronutrients (primarily P/C ratio) dictates mitochondrial functions, cardiometabolomic health and aging.

### Proteins

Proteins are essential macronutrients. They are building blocks of body tissue and can serve as a fuel source. In higher eukaryotes, essential amino acids are taken up through the diet, whereas non-essential amino acids are synthesized *de novo*. For the latter, mitochondria are critical, since they provide oxaloacetate for aspartate and asparagine, as well as 2-oxoglutarate (α-ketoglutarate) for glutamate, glutamine, arginine and proline biosynthesis.

The first step in protein metabolism is to break it into its constituent amino acids. The second step is to break down the amino acids into their constituent parts. This removes the nitrogen or amino group from the amino acids and is called deamination. The carbon skeleton, which is composed of carbon, hydrogen and oxygen, can then be used either for protein synthesis, energy production or converted into glucose by gluconeogenesis in the liver or kidney. Energetically, gluconeogenesis is expensive and therefore the amount of protein converted into glucose is usually small except under conditions of metabolic starvation.

One amino acid that can influence energy production and potentially the fate of mtDNA variants is glutamine (Figure 4). Glutamine is the most abundant amino acid in blood and is second only to glucose as a carbon source for energy production and anabolic processes. Glutamine is a major source of nitrogen for non-essential amino acids, nts and hexosamines [112] and exerts control over the mammalian target of rapamycin (mTOR) signalling [113]. The pathway senses and integrates a variety of environmental cues to regulate organismal growth and homoeostasis and contributes to glutathione synthesis and redox homoeostasis [114]. Mitochondrial glutamine metabolism can follow either oxidative or reductive pathways that occur in response to any mutation or intervention that increases the 2-oxoglutarate/citrate ratio [115,116].

Another amino acid pertinent to the present review is methionine. Grandison et al. [117] found that dietary amino acids are responsible for lifespan shortening and increasing reproduction, but both longevity and fecundity can be maximized when intake of these nutrients is finely tuned. Adding essential amino acids to a restricted diet increased fecundity and decreased lifespan, similar to full feeding, with other nutrients having little or no effect. However, methionine alone increased fecundity as much as full feeding, but without reducing lifespan. Methionine also decreases mitochondrial ROS production [118,119].

### Carbohydrate

Carbohydrates are biological macronutrients consisting of carbon, hydrogen and oxygen. They come in simple forms such as sugars and in complex forms such as starches and fibres. Carbohydrate metabolism denotes the various biochemical processes responsible for the formation, breakdown and interconversion of carbohydrates in living organisms. Typically, carbohydrates can be broken down to produce energy.

Arguably, the most important carbohydrate is glucose, a simple sugar (monosaccharide) that is metabolized by nearly all known organisms. Glycolysis (conversion of glucose into two molecules of pyruvate) occurs in the cytosol of the cell (Figure 2). Pyruvate may cross into mitochondria or be converted into lactate by the enzyme lactate dehydrogenase (LDH). The free energy released in the process of glycolysis is used to form two molecules of NADH and ATP. But, the inner mitochondrial membrane is impermeable to most molecules including NADH. As a consequence, electrons from cytosolic NADH enter mitochondria by shuttles. One of several means of introducing electrons from NADH into the electron transport chain is the malate–aspartate shuttle, which is used in many mammalian tissues including the liver and heart. Another shuttle is the glycerol 3-phosphate shuttle that transfers an electron pair from NADH in the cytoplasm to form FADH_2_ in the mitochondrial matrix. Two lactate shuttle concepts have been proposed as mechanisms to distribute carbohydrate potential energy for oxidation and gluconeogenesis [120]. The intercellular or cell–cell lactate shuttle involves lactate generated and exported from a cell to be taken up and utilized by another cell [120]. The intracellular lactate shuttle hypothesis posits that lactate formed during glycolysis can be continuously used as an energy source within the same cell [120]. The cell–cell lactate shuttle has gained general acceptance; the finer details of the intracellular lactate shuttle continue to be investigated [121].

Pyruvate from glycolysis crosses into mitochondria where it is oxidized to acetyl-CoA and CO_2_ by the pyruvate dehydrogenase complex (Figure 2). When oxygen is present, the mitochondria will undergo aerobic respiration, which leads to the tricarboxylic acid (TCA) cycle. The net gain of high-energy compounds from one cycle is three NADH, one FADH_2_, one GTP; the GTP may subsequently be used to produce ATP (Figure 1).

### Fat

Fats are an important foodstuff for many organisms and can serve both structural and metabolic functions. They are a necessary part of the diet of most heterotrophs but are not required by many insects. In fact, insects metabolize glucose to synthesize lipids and many insecticides target this pathway of lipogenesis. Some fatty acids that are set free by digestion are termed essential because they cannot be synthesized in the body from simpler constituents. Other lipids needed by the body can be synthesized. In mammals, excess energy is stored primarily as triglycerides, which are mobilized when energy demands arise [122].

Fats are categorized according to the number and bonding of the carbon atoms in the aliphatic chain. Saturated fats have no double bonds between the carbons in the chain whereas unsaturated fats have one or more double bonded carbons in the chain. When compared with other macronutrient classes (protein and carbohydrates), fatty acids yield the most ATP on an energy per gram basis by a pathway called β-oxidation. Importantly, β-oxidation of different fats influences the FADH_2_/NADH ratio, which in turn influences the ratio of substrates entering complex I (Figure 1). The shorter the fatty acid, the less FADH_2_ per unit NADH it produces. Short chain fatty acids like C_4_ butyric acid have an FADH_2_/NADH ratio of 0.43 whereas very long chain fatty acids, up at 26 carbons, have an FADH_2_/NADH ratio of approximately 0.49. In mammals, the electron transfer flavoprotein-Q reductase is an additional entry point to the ETS and is important in β-oxidation of fatty acids and catabolism of amino acids and choline [123].

In humans and most other mammals, acetyl-CoA formed in the liver during oxidation of fatty acids can either enter the TCA cycle or undergo conversion to the ‘ketone bodies’ acetone, acetoacetate and D-*β*-hydroxybutyrate for export to other tissues. Acetone is exhaled. Acetoacetate and D-*β*-hydroxybutyrate are transported by blood to tissues where they are converted into acetyl-CoA providing much of the energy required by tissues.

In addition to influencing the FADH_2_/NADH ratio, high-fat diets can reduce the activity and assembly of all ETS complexes to approximately 50%–60% by reducing synthesis of their subunits [124]. A reduction in the activity of ETS complexes is expected to reduce levels of ATP production, which may influence ATP/AMPK signalling. In the next section, we consider the influence of starvation as an ultimate dietary extreme.

### Starvation

In natural populations, the availability of food can be a strong selective force and the ability to survive periods of stress may be an important parameter that influences the frequency of mtDNA haplotypes in nature. Drovetski et al. [125] took advantage of a natural starvation induced die-off in a seabird population [126] to test whether mtDNA haplotypes differ in their probability of being eliminated during such a short-term but marked event. A comparison of the single-dominant mtDNA haplotype frequency between live birds sampled on breeding colonies before and after the die-off showed that fewer individuals harbouring the dominant haplotype died than did carriers of other haplotypes. In this case, the mtDNA appeared to influence survival and it was hypothesized that positive selection acted on the dominant mtDNA haplotype during the die-off.

Higher starvation resistance is expected to be an advantage when suitable food substrates are not continuously available through time and/or space. During fasting, starvation and hibernation, some animals can down-regulate their resting metabolic rate and drastically increase metabolic rate upon food intake [127]. This phenotypic flexibility of mitochondrial energy transduction processes allows king penguin chicks to adjust the cost of their metabolic performance to cope with stochastic parental food provision during the austral winter. Monternier et al. [128] show that the effective efficiency of mitochondrial OXPHOS adjusted to the fasting status of winter king penguin chicks by producing less heat. Mitochondria from re-fed chicks were in turn more ‘thermogenic’, oxidizing more nutrients and thus producing more heat to synthesize the same amount of ATP as fasted chicks. Thus, in this situation, loss of energy through increased proton uncoupling may be disadvantageous during the long cold months. Certainly, it would be intriguing to analyse the pattern of genetic variation in mtDNA encoded genes as compared with nuclear-encoded genes in these king penguins to determine whether selection is acting on the mtDNA genome.

In *Drosophila*, mtDNA haplotype influences starvation resistance [129]. Starvation of both larvae and adults leads to a strong down-regulation of genes involved in mitochondrial translation, respiration, TCA cycle, fatty acid oxidation and mitochondrial transport [130–132]. In larvae, these changes include decreases in ribosome and protein synthesis [133–135] alterations in the storage and metabolism of fat and carbohydrate [135,136]. These alterations are essential for arresting growth and development and maintaining homoeostasis under poor nutrient conditions [134]. Teleman et al. [135] provides evidence in *Drosophila* that Thor is activated under conditions of environmental stress to control fat metabolism. Increasing Thor activity within the context of a whole living animal increases fat accumulation. Conversely, reduced Thor activity leads to an increased rate of fat burn. In combination, these data findings strongly suggest that Thor plays an important role as a regulator of metabolism in *Drosophila*.

In the next section, we consider the influence of diet on complex I mutations as a case study. If mutations in any specific gene were to be influenced by selection, then the frequency of all linked mutations will be affected by the process of genetic hitchhiking [137]. Even the quickly evolving non-coding origin-of-replication region cannot be assumed to have neutral allele frequencies. Experimentally, it is difficult to differentiate those mutations that selection is acting upon from linked mutations. In this regard the lack of a robust system to manipulate metazoan mtDNA is a limitation and support for the development of such systems needs to be encouraged [138]. In parallel, utilization of structural models can be helpful in making predictions of the bioenergetic and functional consequences of specific mutations [45,139–141].

## COMPLEX I MUTATIONS AND DIET AS A CASE STUDY

Here we consider the interactions between diet and ETS complex I and as a case study. First, we briefly review the structure. We then consider a study that reviews the breadth of selection in mtDNA and propose a hypothesis that incorporates an intimate understanding of the structure of complex I, the diet of the organism and mitochondrial functions. We then consider the role of complex I in signalling and suggest that mutations may influence signalling in a context-dependent manner. Next, we provide evidence to suggest that mutations in complex I are differentially influenced by life-history stage in *Drosophila*. Finally, we discuss a dietary modification strategy that may be able to be included as a part of a programme to treat complex I mutations in humans.

Mammalian complex I is one of the largest and most complicated enzymes in the cell. Complex I from *Bos taurus* heart mitochondria has been characterized extensively and contains 44 different subunits (encoded by both the mitochondrial and the nuclear genomes) and nine redox cofactors (a flavin mononucleotide and eight iron–sulphur clusters) [44]. Fourteen subunits are the ‘core’ subunits that are conserved in all complex I enzymes and contain all the mechanistically critical cofactors and structural elements and are sufficient for catalysis [142]. The seven nuclear-encoded core subunits reside in the hydrophilic domain and participate in the oxidation of NADH and transfer of electrons to ubiquinone. The membrane bound hydrophobic domain consists of the four main proton ‘pumps’ that are formed by the seven highly conserved mitochondrial-encoded complex I subunits. In this complex, the movement of four protons accompanies the transfer of two electrons to ubiquinone across the membrane. Proton translocation appears to be driven by conserved charged amino acids that are located along the central axis that traverses the four proton pumps [143,144]. The movement is probably also co-ordinated by a long lateral helix, which is essential for complex I function [145,146].

Complex I may be a repeated target of selection. Following the identification of positively selected sites in Pacific Salmon [140], Garvin et al. [141] searched for signatures of positive selection among the coding mitochondrial genomes of 237 species with a common set of tests. Intriguingly, they provide evidence to support the hypothesis that complex I is a repeated target of positive Darwinian selection in diverse taxa. Notably, these workers identified seasonal availability of food and the utilization of substrates during mitochondrial metabolism as a set of shared traits among taxa where selection was identified. Here we extend their hypothesis and suggest this may result from differential provisioning of the ETS by macronutrients linked with retrograde signalling to the nucleus to indicate the metabolic status of the mitochondrion.

mtDNA mutations in complex I may influence the NAD^+^/NADH ratio by decreasing the conversion of NADH to NAD^+^ and increase ROS levels (Figure 1). Alterations in the NAD^+^/NADH ratio may change the 2-oxogluterate/ citrate ratio shifting the glutamine utilization from oxidative to reductive [115]. A complementary prediction is that increasing the ratio of dietary protein will increase the probability that the TCA cycle will reverse in flow. This is expected to increase citrate production of acetyl-coA and lactate and lipid levels. This hypothesis is supported by data from humans. In approximately 80% of the patients with isolated complex I deficiency high lactate concentrations and elevated lactate/ pyruvate ratios are found, indicating a severely increased NADH/NAD^+^ ratio [147]. NADH feeding electrons into complex I are also expected to release ROS as well as pump protons [76,148]. This may not be deleterious under all circumstances as these molecules are important in cellular signalling. Owusu-Ansah et al. [72] examined *Drosophila* cell lines that contained a complex I mutation and observed that it retarded the cell cycle during the G_1_-S transition. The mechanism involved a specific signalling cascade initiated by ROS and transduced by ASK-1, JNK, FOXO and the *Drosophila* p27 homologue, Dacapo.

A further twist to the identification of functionally significant mutations in complex I is that life-history stages may not be equally affected. In *Drosophila*, complex I mutations may affect larval growth because dietary nutrients are fed through this complex in immaturity. In contrast, it appears that the majority of electrons are provided to complex III in adult *Drosophila* so complex I may be relatively less important. Rechsteiner [149] showed that *Drosophila* LDH and glycerol-3 phosphate dehydrogenase (GPDH) change in activity during development implying that the different life-history stages may have differing reliance on these shuttles. LDH is high in latter instar larvae and low in adults; in contrast, GPDH is low in larvae and high in the emerged adults [149]. High levels of LDH have also been found in ***Phormia*** and ***Chironomus*** but not ***Tenebrio*** larvae [150]. In *Drosophila melanogaster*, three GPDH isozymes (GPDH-1, GPDH-2 and GPDH-3) are characterized and these isozymes originated by alternative splicing from the primary transcript of a single gene [151]. Each isozyme performs a distinct metabolic function: GPDH-1 is involved in the flight muscle metabolism. GPDH-2 and GPDH-3 provide precursor for lipid biosynthesis in the gonads, fat bodies and abdomen.

In humans, complex I dysfunction occurs in a range of cancers [152]. It is also the most common ETS complex causing mitochondrial diseases. For example, mutations in the mitochondrial gene that encodes the complex I subunit NADH dehydrogenase subunit 2 have been shown to be a cause of Leigh syndrome. It has been proposed that dietary modification enabling the bypassing of complex I by providing more reducing equivalents to complex II (Figure 1) may be beneficial in mitochondrial diseases due to complex I deficiency [153]. This concept has been shown to lead to clinical and biochemical improvement in patients with mitochondrial myopathy due to complex I deficiency [153]. Interestingly, one group has tried the opposite approach and treated MELAS with oral nicotinamide, thus attempting to saturate a deficient complex I. Biochemical improvements with reduction in lactate were noted, but no clinical information was provided [154].

## CONCLUSION

A clearer understanding of the link between spatial and/or temporal habitat heterogeneity and mtDNA variation will ultimately result in a broader and more precise understanding of the frequency of specific mtDNA haplotypes in natural populations. This has important implications for (1) adaptation to climate and the environment, especially in the light of climate change, (2) understanding the frequency of specific mutations in populations, (3) predicting the consequences of transferring individuals between geographic localities for pest and biological control and (4) reconstructing the historical movement of species.

Current evidence suggests that the frequency of mtDNA types in natural populations may be influenced by diet. Further, given that dietary macronutrients may change between seasons and life-history stages, diet-induced balancing selection may be a possible mechanism whereby the frequency of haplotypes may be maintained at intermediate frequency in populations. Mechanistically, this may result from differential provisioning of the ETS by macronutrients combined with retrograde signalling to the nucleus to indicate the metabolic status of the mitochondrion.

Future research in this area will need to integrate cellular and organismal approaches to examine mitochondrial functions. Ideally, studies of life-history trade-offs will need to consider and account for not only the rate and efficiency of energy metabolism but also for mitochondrial signalling. The amount of ATP generated per molecule of oxygen consumed can vary significantly between daily feeding and fasting cycles both among and within different tissues of the same individual. As a consequence, it will be necessary to combine subcellular, tissue and whole-organism measurements of metabolism to provide a more robust framework for understanding how the influence of specific mtDNA mutations will influence organismal fecundity, particularly in stressful environmental conditions. For example, a high ADP/O ratio does not necessarily result in high ATP production since this ratio can also be offset by a decrease in oxygen consumption rate [19]); or is it the case that individuals with a relatively low ADP/O ratio are necessarily producing less ATP than those with a higher ADP/O, since this will depend on the rate of work of their mitochondria. Therefore, measuring multiple levels of energetic processes may give a better insight into the energy metabolism, since the rate of ATP generation is dependent on both the rate of oxygen consumption and the efficiency with which that consumed oxygen is used to make ATP.

## Abbreviations

AMPK: AMP-activated protein kinase
ASK-1: apoptosis signal-regulating kinase 1
dFoxo: *Drosophila* forkhead box protein O
ETS: electron transport system
FOXO: transcription factors of the class O forkhead box
GPDH: glycerol-3 phosphate dehydrogenase
JNK: c-Jun N-terminal kinase
KEAP1: Kelch-like ECH-associated protein 1
LDH: lactate dehydrogenase
MELAS: Mitochondrial encephalomyopathy, lactic acidosis, and stroke-like episodes
NAD: Nicotinamide adenine dinucleotide
nDNA: nuclear DNA
NAMPT: nicotinamide phosphoribosyltransferase
NRF2: NF-E2-related factor 2
OXPHOS: oxidative phosphorylation
P/C: protein/carbohydrate
ROS: reactive oxygen species
RTG: retrograde pathway in yeast
SAM: S-adenosyl methionine
SIRT1: Sirtuin (silent mating type information regulation 2 homolog) 1
TCA: tricarboxylic acid

## References

[1] Hedrick P.W. (2006). Genetic polymorphism in heterogeneous environments: the age of genomics. Annu. Rev. Ecol. Evol. Syst..

[2] Bergland A.O., Behrman E.L., O'Brien K.R., Schmidt P.S., Petrov D.A. (2014). Genomic evidence of rapid and stable adaptive oscillations over seasonal time scales in *Drosophila*. PLoS Genet..

[3] Leffler E.M., Gao Z., Pfeifer S., Segurel L., Auton A., Venn O., Bowden R., Bontrop R., Wall J.D., Sella G. (2013). Multiple instances of ancient balancing selection shared between humans and chimpanzees. Science.

[4] Wright S. (1969). Evolution and the Genetics of Populations, Volume 2: The Theory of Gene Frequencies.

[5] Owusu-Ansah E., Song W., Perrimon N. (2013). Muscle mitohormesis promotes longevity via systemic repression of insulin signaling. Cell.

[6] Ballard J.W.O., Pichaud N. (2014). Mitochondrial DNA: More than an evolutionary bystander. Funct. Ecol..

[7] Yun J., Finkel T. (2014). Mitohormesis. Cell Metab..

[8] Epstein C.B., Waddle J.A., Hale W.t., Dave V., Thornton J., Macatee T.L., Garner H.R., Butow R.A. (2001). Genome-wide responses to mitochondrial dysfunction. Mol. Biol. Cell.

[9] Herrera A., Garcia I., Gaytan N., Jones E., Maldonado A., Gilkerson R. (2015). Endangered species: mitochondrial DNA loss as a mechanism of human disease. Front. Biosci..

[10] Shao Z., Graf S., Chaga O.Y., Lavrov D.V. (2006). Mitochondrial genome of the moon jelly *Aurelia aurita* (Cnidaria, Scyphozoa): a linear DNA molecule encoding a putative DNA-dependent DNA polymerase. Gene.

[11] Dong W.G., Song S., Jin D.C., Guo X.G., Shao R. (2014). Fragmented mitochondrial genomes of the rat lice, *Polyplax asiatica* and *Polyplax spinulosa*: intra-genus variation in fragmentation pattern and a possible link between the extent of fragmentation and the length of life cycle. BMC Genomics.

[12] Simpson S.J., Raubenheimer D. (2012). The Nature of Nutrition. A Unifying Framework from Animal Adaptation to Human Obesity.

[13] Robert K.A., Bronikowski A.M. (2010). Evolution of senescence in nature: physiological evolution in populations of garter snake with divergent life histories. Am. Nat..

[14] Salin K., Roussel D., Rey B., Voituron Y. (2012). David and Goliath: a mitochondrial coupling problem?. J. Exp. Zool. A Ecol. Genet. Physiol..

[15] Bottje W.G., Carstens G.E. (2009). Association of mitochondrial function and feed efficiency in poultry and livestock species. J. Anim. Sci..

[16] Katewa S.D., Ballard J.W.O. (2007). Sympatric *Dr**o**sophila simulans* flies with distinct mtDNA show difference in mitochondrial respiration and electron transport. Insect Biochem. Mol. Biol..

[17] Wallace D.C., Fan W. (2009). The pathophysiology of mitochondrial disease as modeled in the mouse. Genes. Dev..

[18] Tuppen H.A., Blakely E.L., Turnbull D.M., Taylor R.W. (2010). Mitochondrial DNA mutations and human disease. Biochim. Biophys. Acta.

[19] Salin K., Luquet E., Rey B., Roussel D., Voituron Y. (2012). Alteration of mitochondrial efficiency affects oxidative balance, development and growth in frog (*Rana te**m**poraria*) tadpoles. J. Exp. Biol..

[20] Hill G.E. (2014). Cellular respiration: the nexus of stress, condition, and ornamentation. Integr. Comp. Biol..

[21] Dillin A., Hsu A.L., Arantes-Oliveira N., Lehrer-Graiwer J., Hsin H., Fraser A.G., Kamath R.S., Ahringer J., Kenyon C. (2002). Rates of behavior and aging specified by mitochondrial function during development. Science.

[22] Roff D.A. (2002). Life History Evolution.

[23] Ballard J.W.O., Dean M.D. (2001). The mitochondrial genome: mutation, selection and recombination. Curr. Opin. Genet. Dev..

[24] Acosta M.L., Sanchez A., Garcia F., Contreras A., Molina E. (2007). Analysis of kinetic, stoichiometry and regulation of glucose and glutamine metabolism in hybridoma batch cultures using logistic equations. Cytotechnology.

[25] Salin K., Auer S.K., Rey B., Selman C., Metcalfe N.B. (2015). Variation in the link between oxygen consumption and ATP production, and its relevance for animal performance. Proc. Biol. Sci..

[26] Lopez-Crisosto C., Bravo-Sagua R., Rodriguez-Pena M., Mera C., Castro P.F., Quest A.F., Rothermel B.A., Cifuentes M., Lavandero S. (2015). ER-to-mitochondria miscommunication and metabolic diseases. Biochim. Biophys. Acta.

[27] Buttgereit F., Brand M.D. (1995). A hierarchy of ATP-consuming processes in mammalian cells. Biochem. J..

[28] Ballard J.W.O., Whitlock M.C. (2004). The incomplete natural history of mitochondria. Mol. Ecol..

[29] Stearns S.C. (1992). The Evolution of Life Histories.

[30] Nicholls D.G., Ferguson S.J. (2002). Bioenergetics.

[31] Brand M.D. (2000). Uncoupling to survive? The role of mitochondrial inefficiency in ageing. Exp. Gerontol..

[32] Brand M.D. (2005). The efficiency and plasticity of mitochondrial energy transduction. Biochem. Soc. Trans..

[33] Gershoni M., Templeton A.R., Mishmar D. (2009). Mitochondrial bioenergetics as a major motive force of speciation. Bioessays.

[34] Evans M.L., Bernatchez L. (2012). Oxidative phosphorylation gene transcription in whitefish species pairs reveals patterns of parallel and nonparallel physiological divergence. J. Evol. Biol..

[35] Chance B., Williams G.R. (1956). The respiratory chain and oxidative phosphorylation. Adv. Enzymol. Relat. Subj. Biochem..

[36] Horan M.P., Pichaud N., Ballard J.W.O. (2012). Review: quantifying mitochondrial dysfunction in complex diseases of aging. J. Gerontol. A Biol. Sci. Med. Sci..

[37] Stillwell W., Jenski L.J., Crump F.T., Ehringer W. (1997). Effect of docosahexaenoic acid on mouse mitochondrial membrane properties. Lipids.

[38] Yu L., Fink B.D., Herlein J.A., Oltman C.L., Lamping K.G., Sivitz W.I. (2014). Dietary fat, fatty acid saturation and mitochondrial bioenergetics. J. Bioenerg. Biomembr..

[39] Mishmar D., Ruiz-Pesini E., Golik P., Macaulay V., Clark A.G., Hosseini S., Brandon M., Easley K., Chen E., Brown M.D. (2003). Natural selection shaped regional mtDNA variation in humans. Proc. Natl. Acad. Sci. U.S.A..

[40] Ruiz-Pesini E., Mishmar D., Brandon M., Procaccio V., Wallace D.C. (2004). Effects of purifying and adaptive selection on regional variation in human mtDNA. Science.

[41] Elson J.L., Turnbull D.M., Howell N. (2004). Comparative genomics and the evolution of human mitochondrial DNA: assessing the effects of selection. Am. J. Hum. Genet..

[42] Amo T., Brand M.D. (2007). Were inefficient mitochondrial haplogroups selected during migrations of modern humans? A test using modular kinetic analysis of coupling in mitochondria from cybrid cell lines. Biochem. J..

[43] Sun C., Kong Q.P., Zhang Y.P. (2007). The role of climate in human mitochondrial DNA evolution: a reappraisal. Genomics.

[44] Vinothkumar K.R., Zhu J., Hirst J. (2014). Architecture of mammalian respiratory complex I. Nature.

[45] Melvin R.G., Katewa S.D., Ballard J.W.O. (2008). A candidate complex approach to study functional mitochondrial DNA changes: sequence variation and quaternary structure modeling of *Drosophila simulans* cytochrome c oxidase. J. Mol. Evol..

[46] Huttemann M., Jaradat S., Grossman L.I. (2003). Cytochrome c oxidase of mammals contains a testes-specific isoform of subunit VIb–the counterpart to testes-specific cytochrome c?. Mol. Reprod. Dev..

[47] Blaza J.N., Serreli R., Jones A.J., Mohammed K., Hirst J. (2014). Kinetic evidence against partitioning of the ubiquinone pool and the catalytic relevance of respiratory-chain supercomplexes. Proc. Natl. Acad. Sci. U.S.A..

[48] Jia W., Higgs P.G. (2008). Codon usage in mitochondrial genomes: distinguishing context-dependent mutation from translational selection. Mol. Biol. Evol..

[49] Duvezin-Caubet S., Caron M., Giraud M.F., Velours J., di Rago J.P. (2003). The two rotor components of yeast mitochondrial ATP synthase are mechanically coupled by subunit delta. Proc. Natl. Acad. Sci. U.S.A..

[50] Guan M.X., Fischel-Ghodsian N., Attardi G. (1996). Biochemical evidence for nuclear gene involvement in phenotype of non-syndromic deafness associated with mitochondrial 12S rRNA mutation. Hum. Mol. Genet..

[51] Konovalova S., Tyynismaa H. (2013). Mitochondrial aminoacyl-tRNA synthetases in human disease. Mol. Genet. Metab..

[52] Scarpulla R.C. (2008). Transcriptional paradigms in mammalian mitochondrial biogenesis and function. Physiol. Rev..

[53] Holt I.J., Reyes A. (2012). Human mitochondrial DNA replication. Cold Spring Harb. Perspect. Biol..

[54] Mercer T.R., Neph S., Dinger M.E., Crawford J., Smith M.A., Shearwood A.M., Haugen E., Bracken C.P., Rackham O., Stamatoyannopoulos J.A. (2011). The human mitochondrial transcriptome. Cell.

[55] Zhu C.T., Ingelmo P., Rand D.M. (2014). GxGxE for lifespan in *Drosophila*: mitochondrial, nuclear, and dietary interactions that modify longevity. PLoS Genet..

[56] Arnqvist G., Dowling D.K., Eady P., Gay L., Tregenza T., Tuda M., Hosken D.J. (2010). Genetic architecture of metabolic rate: environment specific epistasis between mitochondrial and nuclear genes in an insect. Evolution.

[57] Dowling D.K., Friberg U., Lindell J. (2008). Evolutionary implications of non-neutral mitochondrial genetic variation. Trends Ecol. Evol..

[58] Willett C.S., Burton R.S. (2001). Viability of cytochrome c genotypes depends on cytoplasmic backgrounds in *Tigriopus californicus*. Evolution.

[59] Bellizzi D., D'Aquila P., Giordano M., Montesanto A., Passarino G. (2012). Global DNA methylation levels are modulated by mitochondrial DNA variants. Epigenomics.

[60] Kelly R.D., Rodda A.E., Dickinson A., Mahmud A., Nefzger C.M., Lee W., Forsythe J.S., Polo J.M., Trounce I.A., McKenzie M. (2013). Mitochondrial DNA haplotypes define gene expression patterns in pluripotent and differentiating embryonic stem cells. Stem Cells.

[61] Waterland R.A., Kellermayer R., Laritsky E., Rayco-Solon P., Harris R.A., Travisano M., Zhang W., Torskaya M.S., Zhang J., Shen L. (2010). Season of conception in rural gambia affects DNA methylation at putative human metastable epialleles. PLoS Genet.

[62] Christie J.S., Picornell A., Moya A., Ramon M.M., Castro J.A. (2011). Mitochondrial DNA effects on fitness in *Drosophila subobscura*. Heredity.

[63] Ballard J.W.O., James A.C. (2004). Differential fitness of mitochondrial DNA in perturbation cage studies correlates with global abundance and population history in *Drosophila simulans*. Proc. Biol. Sci..

[64] James A.C., Ballard J.W.O. (2003). Mitochondrial genotype affects fitness in *Drosophila simulans*. Genetics.

[65] Liu Z., Butow R.A. (2006). Mitochondrial retrograde signaling. Annu. Rev. Genet..

[66] Ballard J.W.O., Melvin R.G. (2011). Early life benefits and later life costs of a two amino acid deletion in *Drosophila simulans*. Evolution.

[67] Rothermel B.A., Shyjan A.W., Etheredge J.L., Butow R.A. (1995). Transactivation by Rtg1p, a basic helix-loop-helix protein that functions in communication between mitochondria and the nucleus in yeast. J. Biol. Chem..

[68] Rothermel B.A., Thornton J.L., Butow R.A. (1997). Rtg3p, a basic helix-loop-helix/leucine zipper protein that functions in mitochondrial-induced changes in gene expression, contains independent activation domains. J. Biol. Chem..

[69] Zhang F., Pracheil T., Thornton J., Liu Z. (2013). Adenosine triphosphate (ATP) is a candidate signaling molecule in the mitochondria-to-nucleus retrograde response pathway. Genes.

[70] Heddi A., Stepien G., Benke P.J., Wallace D.C. (1999). Coordinate induction of energy gene expression in tissues of mitochondrial disease patients. J. Biol. Chem..

[71] Pichaud N., Messmer M., Correa C.C., Ballard J.W.O. (2013). Diet influences the intake target and mitochondrial functions of *Drosophila melanogaster* males. Mitochondrion.

[72] Owusu-Ansah E., Yavari A., Mandal S., Banerjee U. (2008). Distinct mitochondrial retrograde signals control the G1-S cell cycle checkpoint. Nat. Genet..

[73] Ballard J.W.O., Melvin R.G., Lazarou M., Clissold F.J., Simpson S.J. (2010). Cost of a naturally occurring two-amino acid deletion in cytochrome c oxidase subunit 7A in *Drosophila simulans*. Am. Nat..

[74] Payne B.A., Chinnery P.F. (2015). Mitochondrial dysfunction in aging: Much progress but many unresolved questions. Biochim. Biophys. Acta.

[75] Stone J.R., Yang S. (2006). Hydrogen peroxide: a signaling messenger. Antioxid. Redox. Signal..

[76] Miwa S., St-Pierre J., Partridge L., Brand M.D. (2003). Superoxide and hydrogen peroxide production by *Drosophila* mitochondria. Free Radic. Biol. Med..

[77] Quinlan C.L., Orr A.L., Perevoshchikova I.V., Treberg J.R., Ackrell B.A., Brand M.D. (2012). Mitochondrial complex II can generate reactive oxygen species at high rates in both the forward and reverse reactions. J. Biol. Chem..

[78] Boveris A., Chance B. (1973). The mitochondrial generation of hydrogen peroxide. General properties and effect of hyperbaric oxygen. Biochem. J..

[79] Fridovich I. (2004). Mitochondria: are they the seat of senescence?. Aging Cell.

[80] Murphy M.P. (2009). How mitochondria produce reactive oxygen species. Biochem. J..

[81] D'Autreaux B., Toledano M.B. (2007). ROS as signalling molecules: mechanisms that generate specificity in ROS homeostasis. Nat. Rev. Mol. Cell Biol..

[82] Taguchi K., Motohashi H., Yamamoto M. (2011). Molecular mechanisms of the Keap1-Nrf2 pathway in stress response and cancer evolution. Genes Cells.

[83] Brunet A., Sweeney L.B., Sturgill J.F., Chua K.F., Greer P.L., Lin Y., Tran H., Ross S.E., Mostoslavsky R., Cohen H.Y. (2004). Stress-dependent regulation of FOXO transcription factors by the SIRT1 deacetylase. Science.

[84] Teleman A.A., Hietakangas V., Sayadian A.C., Cohen S.M. (2008). Nutritional control of protein biosynthetic capacity by insulin via Myc in *Drosophila*. Cell Metab..

[85] Sun F., Dai C., Xie J., Hu X. (2012). Biochemical issues in estimation of cytosolic free NAD/NADH ratio. PLoS One.

[86] Dolle C., Rack J.G., Ziegler M. (2013). NAD and ADP-ribose metabolism in mitochondria. FEBS J..

[87] Stein L.R., Imai S. (2012). The dynamic regulation of NAD metabolism in mitochondria. Trends Endocrinol. Metab..

[88] Nakagawa T., Lomb D.J., Haigis M.C., Guarente L. (2009). SIRT5 deacetylates carbamoyl phosphate synthetase 1 and regulates the urea cycle. Cell.

[89] Chen D., Bruno J., Easlon E., Lin S.J., Cheng H.L., Alt F.W., Guarente L. (2008). Tissue-specific regulation of SIRT1 by calorie restriction. Genes Dev..

[90] Yang H., Yang T., Baur J.A., Perez E., Matsui T., Carmona J.J., Lamming D.W., Souza-Pinto N.C., Bohr V.A., Rosenzweig A. (2007). Nutrient-sensitive mitochondrial NAD^+^ levels dictate cell survival. Cell.

[91] Canto C., Jiang L.Q., Deshmukh A.S., Mataki C., Coste A., Lagouge M., Zierath J.R., Auwerx J. (2010). Interdependence of AMPK and SIRT1 for metabolic adaptation to fasting and exercise in skeletal muscle. Cell Metab..

[92] Yoshino J., Mills K.F., Yoon M.J., Imai S. (2011). Nicotinamide mononucleotide, a key NAD(+) intermediate, treats the pathophysiology of diet- and age-induced diabetes in mice. Cell Metab..

[93] Iacobazzi V., Castegna A., Infantino V., Andria G. (2013). Mitochondrial DNA methylation as a next-generation biomarker and diagnostic tool. Mol. Genet. Metab..

[94] Byun H.M., Baccarelli A.A. (2014). Environmental exposure and mitochondrial epigenetics: study design and analytical challenges. Hum. Genet..

[95] Maresca A., Zaffagnini M., Caporali L., Carelli V., Zanna C. (2015). DNA methyltransferase 1 mutations and mitochondrial pathology: is mtDNA methylated?. Front Genet..

[96] Bellizzi D., D'Aquila P., Scafone T., Giordano M., Riso V., Riccio A., Passarino G. (2013). The control region of mitochondrial DNA shows an unusual CpG and non-CpG methylation pattern. DNA Res..

[97] Feng S., Xiong L., Ji Z., Cheng W., Yang H. (2012). Correlation between increased ND2 expression and demethylated displacement loop of mtDNA in colorectal cancer. Mol. Med. Rep..

[98] Pirola C.J., Gianotti T.F., Burgueno A.L., Rey-Funes M., Loidl C.F., Mallardi P., Martino J.S., Castano G.O., Sookoian S. (2013). Epigenetic modification of liver mitochondrial DNA is associated with histological severity of nonalcoholic fatty liver disease. Gut.

[99] Chen H., Dzitoyeva S., Manev H. (2012). Effect of valproic acid on mitochondrial epigenetics. Eur. J. Pharmacol..

[100] Wong M., Gertz B., Chestnut B.A., Martin L.J. (2013). Mitochondrial DNMT3A and DNA methylation in skeletal muscle and CNS of transgenic mouse models of ALS. Front Cell Neurosci.

[101] Wallace D.C., Fan W. (2010). Energetics, epigenetics, mitochondrial genetics. Mitochondrion.

[102] Christensen K.E., MacKenzie R.E. (2006). Mitochondrial one-carbon metabolism is adapted to the specific needs of yeast, plants and mammals. Bioessays.

[103] Tobi E.W., Goeman J.J., Monajemi R., Gu H., Putter H., Zhang Y., Slieker R.C., Stok A.P., Thijssen P.E., Muller F. (2014). DNA methylation signatures link prenatal famine exposure to growth and metabolism. Nat. Commun..

[104] Radford E.J., Ito M., Shi H., Corish J.A., Yamazawa K., Isganaitis E., Seisenberger S., Hore T.A., Reik W., Erkek S. (2014). *In utero* effects. *In utero* undernourishment perturbs the adult sperm methylome and intergenerational metabolism. Science.

[105] Li C.C., Young P.E., Maloney C.A., Eaton S.A., Cowley M.J., Buckland M.E., Preiss T., Henstridge D.C., Cooney G.J., Febbraio M.A. (2013). Maternal obesity and diabetes induces latent metabolic defects and widespread epigenetic changes in isogenic mice. Epigenetics.

[106] Buescher J.L., Musselman L.P., Wilson C.A., Lang T., Keleher M., Baranski T.J., Duncan J.G. (2013). Evidence for transgenerational metabolic programming in *Drosophila*. Dis. Model Mech..

[107] Suarez R.K., Lighton J.R., Moyes C.D., Brown G.S., Gass C.L., Hochachka P.W. (1990). Fuel selection in rufous hummingbirds: ecological implications of metabolic biochemistry. Proc. Natl. Acad. Sci. U.S.A..

[108] Welch K.C., Altshuler D.L., Suarez R.K. (2007). Oxygen consumption rates in hovering hummingbirds reflect substrate-dependent differences in P/O ratios: carbohydrate as a ‘premium fuel’. J. Exp. Biol..

[109] Moreno-Sanchez R., Hernandez-Esquivel L., Rivero-Segura N.A., Marin-Hernandez A., Neuzil J., Ralph S.J., Rodriguez-Enriquez S. (2013). Reactive oxygen species are generated by the respiratory complex II–evidence for lack of contribution of the reverse electron flow in complex I. FEBS J..

[110] Lee K.P., Simpson S.J., Clissold F.J., Brooks R., Ballard J.W.O., Taylor P.W., Soran N., Raubenheimer D. (2008). Lifespan and reproduction in *Drosophila*: new insights from nutritional geometry. Proc. Natl. Acad. Sci. U.S.A..

[111] Solon-Biet S.M., McMahon A.C., Ballard J.W.O., Ruohonen K., Wu L.E., Cogger V.C., Warren A., Pichaud N., Melvin R.G., Gokarn R. (2014). The ratio of macronutrients, not caloric intake, dictates cardiometabolic health, aging and longevity in *ad libitum*-fed mice. Cell Metab..

[112] Hensley C.T., Wasti A.T., DeBerardinis R.J. (2013). Glutamine and cancer: cell biology, physiology, and clinical opportunities. J. Clin. Invest..

[113] Nicklin P., Bergman P., Zhang B., Triantafellow E., Wang H., Nyfeler B., Yang H., Hild M., Kung C., Wilson C. (2009). Bidirectional transport of amino acids regulates mTOR and autophagy. Cell.

[114] Le A., Lane A.N., Hamaker M., Bose S., Gouw A., Barbi J., Tsukamoto T., Rojas C.J., Slusher B.S., Zhang H. (2012). Glucose-independent glutamine metabolism via TCA cycling for proliferation and survival in B cells. Cell Metab..

[115] Fendt S.M., Bell E.L., Keibler M.A., Olenchock B.A., Mayers J.R., Wasylenko T.M., Vokes N.I., Guarente L., Vander Heiden M.G., Stephanopoulos G. (2013). Reductive glutamine metabolism is a function of the alpha-ketoglutarate to citrate ratio in cells. Nat. Commun..

[116] Holleran A.L., Briscoe D.A., Fiskum G., Kelleher J.K. (1995). Glutamine metabolism in AS-30D hepatoma cells. Evidence for its conversion into lipids via reductive carboxylation. Mol. Cell. Biochem..

[117] Grandison R.C., Piper M.D., Partridge L. (2009). Amino-acid imbalance explains extension of lifespan by dietary restriction in *Drosophila*. Nature.

[118] Sanz A., Caro P., Ayala V., Portero-Otin M., Pamplona R., Barja G. (2006). Methionine restriction decreases mitochondrial oxygen radical generation and leak as well as oxidative damage to mitochondrial DNA and proteins. FASEB J..

[119] Caro P., Gomez J., Sanchez I., Naudi A., Ayala V., Lopez-Torres M., Pamplona R., Barja G. (2009). Forty percent methionine restriction decreases mitochondrial oxygen radical production and leak at complex I during forward electron flow and lowers oxidative damage to proteins and mitochondrial DNA in rat kidney and brain mitochondria. Rejuvenation Res..

[120] Brooks G.A. (1998). Mammalian fuel utilization during sustained exercise. Comp. Biochem. Physiol. B Biochem. Mol. Biol..

[121] Kane D.A. (2014). Lactate oxidation at the mitochondria: a lactate-malate-aspartate shuttle at work. Front. Neurosci..

[122] Nakamura M.T., Yudell B.E., Loor J.J. (2014). Regulation of energy metabolism by long-chain fatty acids. Prog. Lipid. Res..

[123] Ruzicka F.J., Beinert H. (1977). A new iron-sulfur flavoprotein of the respiratory chain. A component of the fatty acid beta oxidation pathway. J. Biol. Chem..

[124] Garcia-Ruiz I., Solis-Munoz P., Fernandez-Moreira D., Grau M., Colina F., Munoz-Yague T., Solis-Herruzo J.A. (2014). High-fat diet decreases activity of the oxidative phosphorylation complexes and causes nonalcoholic steatohepatitis in mice. Dis. Model Mech..

[125] Drovetski S.V., Kitaysky A.S., Mode N.A., Zink R.M., Iqbal U., Barger C. (2012). mtDNA haplotypes differ in their probability of being eliminated by a mass die-off in an abundant seabird. Heredity.

[126] Piatt J.F., Van Pelt T.I. (1997). Mass-mortality of Guillemots (*Uria aalge*) in the Gulf of Alaska in 1993. Mar. Pollut. Bull..

[127] Secor S.M. (2009). Specific dynamic action: a review of the postprandial metabolic response. J. Comp. Physiol. B..

[128] Monternier P.A., Marmillot V., Rouanet J.L., Roussel D. (2014). Mitochondrial phenotypic flexibility enhances energy savings during winter fast in king penguin chicks. J. Exp. Biol..

[129] Ballard J.W.O., Melvin R.G., Katewa S.D., Maas K. (2007). Mitochondrial DNA variation is associated with measurable differences in life-history traits and mitochondrial metabolism in *Drosophila simulans*. Evolution.

[130] Arbeitman M.N., Furlong E.E., Imam F., Johnson E., Null B.H., Baker B.S., Krasnow M.A., Scott M.P., Davis R.W., White K.P. (2002). Gene expression during the life cycle of *Drosophila melanogaster*. Science.

[131] White K.P., Rifkin S.A., Hurban P., Hogness D.S. (1999). Microarray analysis of *Drosophila* development during metamorphosis. Science.

[132] Gershman B., Puig O., Hang L., Peitzsch R.M., Tatar M., Garofalo R.S. (2007). High-resolution dynamics of the transcriptional response to nutrition in *Drosophila*: a key role for dFOXO. Physiol. Genomics.

[133] Grewal S.S., Evans J.R., Edgar B.A. (2007). *Dr**o**sophila* TIF-IA is required for ribosome synthesis and cell growth and is regulated by the TOR pathway. J. Cell Biol..

[134] Tettweiler G., Miron M., Jenkins M., Sonenberg N., Lasko P.F. (2005). Starvation and oxidative stress resistance in *Drosophila* are mediated through the eIF4E-binding protein, d4E-BP. Genes Dev..

[135] Teleman A.A., Chen Y.W., Cohen S.M. (2005). 4E-BP functions as a metabolic brake used under stress conditions but not during normal growth. Genes Dev..

[136] Britton J.S., Lockwood W.K., Li L., Cohen S.M., Edgar B.A. (2002). Drosophila's insulin/PI3-kinase pathway coordinates cellular metabolism with nutritional conditions. Dev. Cell.

[137] Smith J.M., Haigh J. (1974). The hitch-hiking effect of a favourable gene. Genet. Res..

[138] Xu H., DeLuca S.Z., O'Farrell P.H. (2008). Manipulating the metazoan mitochondrial genome with targeted restriction enzymes. Science.

[139] Horan M.P., Rumbley J.N., Melvin R.G., Le Couteur D.G., Ballard J.W.O. (2013). Quaternary protein modeling to predict the function of DNA variation found in human mitochondrial cytochrome c oxidase. J. Hum. Genet..

[140] Garvin M.R., Bielawski J.P., Gharrett A.J. (2011). Positive Darwinian selection in the piston that powers proton pumps in complex I of the mitochondria of Pacific salmon. PLoS One.

[141] Garvin M.R., Bielawski J.P., Sazanov L.A., Gharrett A.J. (2015). Review and meta-analysis of natural selection in mitochondrial complex I in metazoans. J. Zoolog. Syst. Evol. Res..

[142] Efremov R.G., Sazanov L.A. (2011). Respiratory complex I: ‘steam engine’ of the cell?. Curr. Opin. Struct. Biol..

[143] Baradaran R., Berrisford J.M., Minhas G.S., Sazanov L.A. (2013). Crystal structure of the entire respiratory complex I. Nature..

[144] Hirst J. (2013). Mitochondrial complex I. Annu Rev Biochem..

[145] Efremov R.G., Sazanov L.A. (2011). Structure of the membrane domain of respiratory complex I. Nature.

[146] Torres-Bacete J., Sinha P.K., Matsuno-Yagi A., Yagi T. (2011). Structural contribution of C-terminal segments of NuoL (ND5) and NuoM (ND4) subunits of complex I from *Escherichia coli*. J. Biol. Chem..

[147] Munnich A., Rotig A., Chretien D., Saudubray J.M., Cormier V., Rustin P. (1996). Clinical presentations and laboratory investigations in respiratory chain deficiency. Eur. J. Pediatr..

[148] Hirst J., King M.S., Pryde K.R. (2008). The production of reactive oxygen species by complex I. Biochem. Soc. Trans..

[149] Rechsteiner M.C. (1970). Drosophila lactate dehydrogenase and alpha-glycerolphosphate dehydrogenase: distribution and change in activity during development. J. Insect. Physiol..

[150] Zebe E.C., McShan W.H. (1957). Lactic and alpha-glycerophosphate dehydrogenases in insects. J. Gen. Physiol..

[151] Cook J.L., Bewley G.C., Shaffer J.B. (1988). Drosophila sn-glycerol-3-phosphate dehydrogenase isozymes are generated by alternate pathways of RNA processing resulting in different carboxyl-terminal amino acid sequences. J. Biol. Chem..

[152] Iommarini L., Calvaruso M.A., Kurelac I., Gasparre G., Porcelli A.M. (2013). Complex I impairment in mitochondrial diseases and cancer: parallel roads leading to different outcomes. Int. J. Biochem. Cell Biol..

[153] Roef M.J., de Meer K., Reijngoud D.J., Straver H.W., de Barse M., Kalhan S.C., Berger R. (2002). Triacylglycerol infusion improves exercise endurance in patients with mitochondrial myopathy due to complex I deficiency. Am. J. Clin. Nutr..

[154] Majamaa K., Rusanen H., Remes A., Hassinen I.E. (1997). Metabolic interventions against complex I deficiency in MELAS syndrome. Mol. Cell. Biochem..

